# An unusual mechanism of spinal cord injury due to active neck stretching and its functional implications

**DOI:** 10.1002/ccr3.2831

**Published:** 2020-04-12

**Authors:** Sami Ullah, Ahmad Zaheer Qureshi, Sherif Samir Tantawy, Yazid Antar AlJaizani

**Affiliations:** ^1^ Department of Physical Medicine & Rehabilitation King Fahad Medical City Riyadh Saudi Arabia

**Keywords:** rehabilitation, Saudi Arabia, spinal cord injury, spinal manipulation

## Abstract

Spinal cord injury without radiological abnormality is a rare entity and has not been reported to occur secondary to active neck stretching. The case report highlights the possible mechanisms of injury and functional outcomes of multidisciplinary rehabilitation.

## Graphical abstract

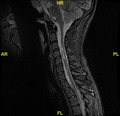

## 1 INTRODUCTION

A young individual developed spinal contusion without radiological evidence of vertebral column damage after active neck stretching and was diagnosed with spinal cord injury without radiological abnormality (SCIWORA). The case is reported due to a rare etiology of SCIWORA and highlights the functional outcomes of this unique presentation.

Trauma to the spinal cord is a frequent cause of paraplegia and tetraplegia. Every year, around 40 million people worldwide suffer from spinal cord injury (SCI) with most of them occurring at a young age.^1^ Saudi Arabia has one of the highest rates of SCI in the world which are most commonly occurring secondary to motor vehicle accidents (MVA), falls, or penetrating injuries.^2^ Usually, injuries to the spinal cord are associated with damage to other structures surrounding the spinal cord such as vertebral fractures, dislocations, disk protrusions, and disruption of ligamentous apparatus. Although traumatic injury to the spinal cord without osseous damage is well documented in the pediatric population, it is rarely reported in adults.^3^ Traumatic SCI demonstrating no osseous abnormalities of the spine is known as SCI without radiological abnormality (SCIWORA). This term is defined as “clinical symptoms of traumatic myelopathy where no radiographic or computed tomographic features of spinal fracture or instability can be found.”^4^ The term SCIWORA mainly accounts for the absence of fractures or instability with or without radiological evidence of neural injury. Herein, we present a rare clinical presentation of spinal cord contusion without radiological evidence of fracture or instability that occurred as a consequence of active neck stretching. Our case is intended to highlight a rare mechanism of injury leading to SCIWORA which has not been reported in the literature before.

## 2 CASE REPORT

**FIGURE 1:**
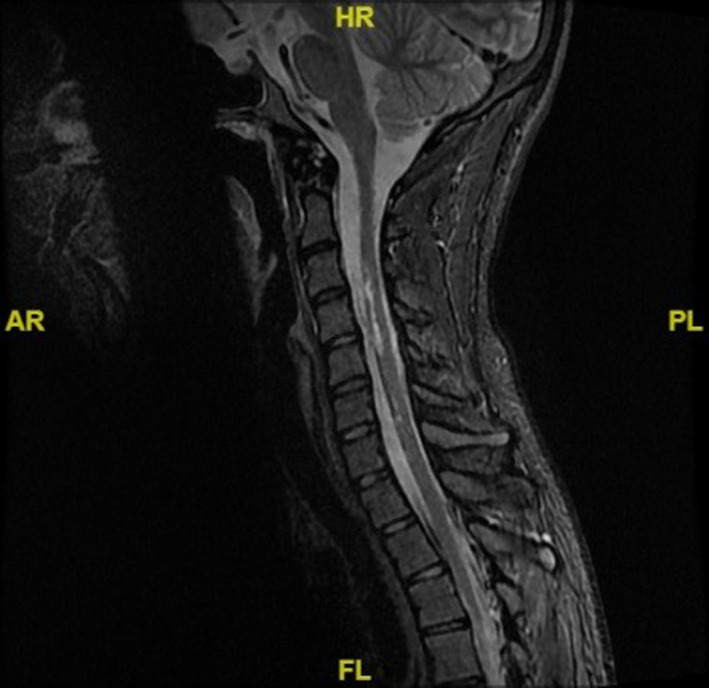
MRI Sagittal T‐2 image of cervical spine showing multilevel (C 2‐ C6) spinal contusion without evidence of spinal fracture or instability

An 18‐year‐old male patient developed neck pain shortly after stretching his neck. He was sitting comfortably on a chair for prolonged duration and extended his neck in a sustained stretch for about 4‐5 seconds. This was followed by abrupt rotational movements of the neck which he used to do typically when he felt tired from prolonged sitting. There was no prior neck pain; however, he started to have pain on the back of the neck radiating down the upper extremities associated with tingling and numbness. It later progressed to lower extremities followed by weakness in all four limbs over the next 24 hours. On arrival to the local hospital, he was diagnosed with cervical cord contusion on MRI, with no other abnormalities (Figure 1). Patient was treated conservatively for 3 days and was discharged home with outpatient physical therapy till he was admitted to our specialized SCI rehabilitation unit for comprehensive intensive rehabilitation program after 1 month of onset. Based on American Spinal Cord Injury Association (ASIA) scale evaluation, patient was assessed to have SCI C 5 ASIA D at the time of admission to rehabilitation. Sensory examination was normal in all segments including sacral segments S4‐S5. Voluntary anal tone was present. Motor assessment of key muscles on the right side was 4/5 in the upper limb and 5/5 in the lower limb; however, the motor assessment for left upper limb was [C5,C6,C7,C8,T1 = 2/5] and for the left lower limb was [L2,L3,L4,L5,S1 = 4/5]. Passive range of motion was within normal limits across all joints, and examination was unremarkable for joint laxity or connective tissue disorders. He had Modified Ashworth Scale grade I spasticity in left ankle planter flexors. This was associated with foot inversion during swing phase of gait likely secondary to dynamic increase in tone of ankle invertors leading to ankle instability during initial foot contact and stance phase. Truncal stability evaluation and hip bridging endurance test showed core muscle weakness of the trunk. Consequently, he had fair dynamic sitting balance and demonstrated posterior pelvic tilt associated with increase in thoracolumbar kyphosis during standing, which was more evident during initial gait assessment. His preadmission physical therapy was limited to strength training in the extremities, which may have attributed to truncal muscle atrophy and deconditioning as gait and core muscle strength training was not started till the time of admission to rehabilitation. This could explain his apprehension of fall and need for maximum assistance in ambulation during initial assessment. He was continent to bladder and bowel with no postvoid residuals or episodes of incontinence.

**FIGURE 2:**
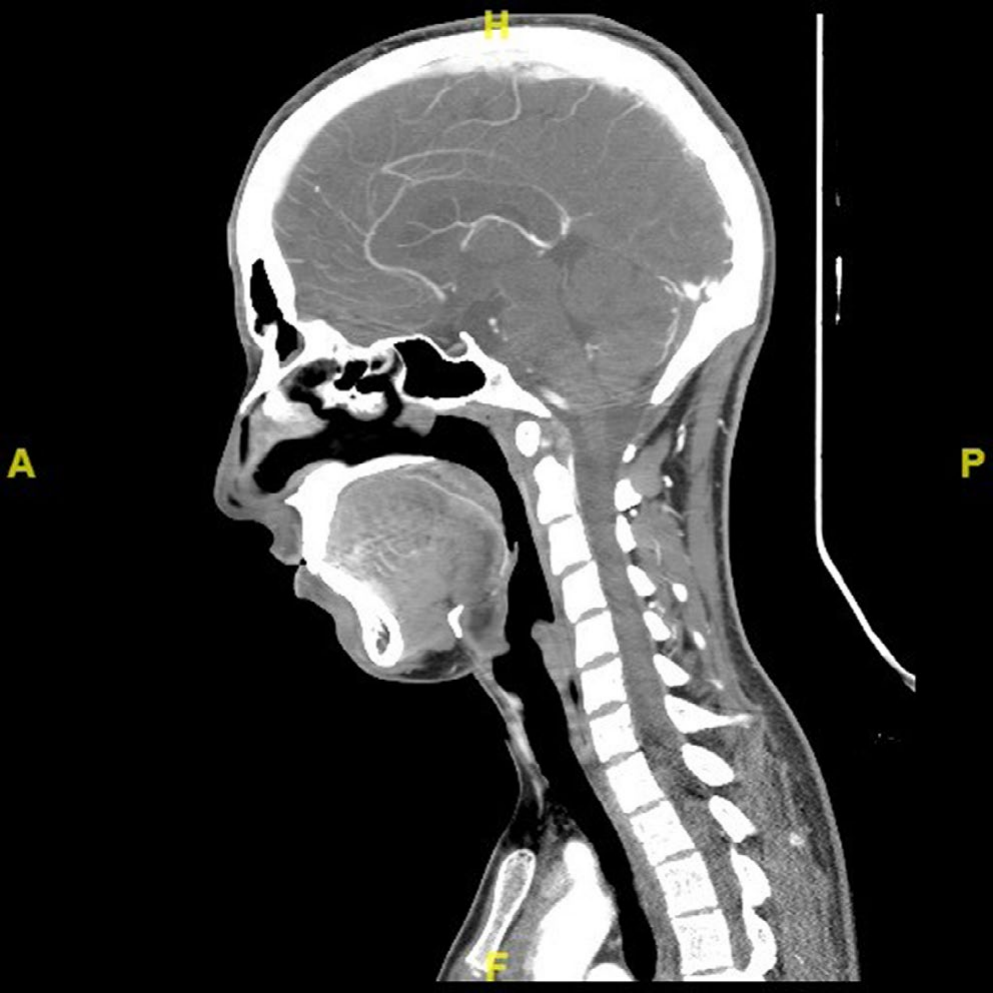
CT angiogram of the neck vessels and intracranial circulation with no significant findings

Neurology and rheumatology teams were involved to look for vascular causes of spinal contusion, and extensive workup including angiography (Figure 2) ruled out any vascular, immunological, or rheumatological etiology. Patient underwent 4 weeks of comprehensive inpatient rehabilitation program and received 3 hours per day rehabilitation services from occupational therapy, physical therapy, nursing, recreational therapy, and rehabilitation medicine specialists. Physical therapist focused on treatment of motor control, spasticity, strength and balance training, transfers, ambulation and stair climbing, whereas the occupational therapist focused on maximizing the functional independence self‐care, equipment provision, and home modifications. The functional goals of rehabilitation were to improve independence in upper body activities, transfers, and gait. His ankle spasticity was treated with stretching, range of motion exercises, and ankle‐foot orthosis was provided to improve ankle stability during gait. On discharge from inpatient rehabilitation unit, he achieved independence in feeding and transfers. He was modified independent in feeding and dressing, and only required setup in bathing and toileting. His Functional Independence Measure (FIM) score was 83 at the time of admission and 94 at the time of discharge with a FIM gain of 11. He was counseled on ergonomics and was instructed to avoid sudden strenuous neck stretching maneuvers as it was the most likely etiology of spinal contusion in his case.

## 3 DISCUSSION

Active neck stretching has not been reported in the literature as a cause of SCI. Passive neck stretching or cervical manipulation in a patient can result in plexopathy or radiculopathy, but its association with a cord injury is a rare entity.^5^ Such injuries are reportedly associated with premorbid structural abnormalities of the spine. For example, spinal manipulation therapy resulted in SCI in a 68‐year‐old man with nontraumatic acute herniated nucleus pulposus (HNP) and a 61‐year‐old woman with neck pain who had previous ossification in posterior longitudinal ligament.^6^, ^7^ Moreover, SCIWORA is generally associated with trauma involving impact or blunt injuries and in rare cases passive stretching or manipulation, as mentioned above. Our case is unique in sense, as the neck movements were actively done by the patient himself. In Saudi Arabia, where MVA is the most common cause of SCI, the absence of a direct hit or impact trauma rendered the need of immunological and vascular work to rule out nontraumatic causes of SCI in this young individual.^8^ The possibility of a systemic illness was less as the symptoms started immediately after an obvious event of neck stretching. The absence of any osseous or soft tissue abnormality on the imaging raised the possibility of a vascular event during the active neck stretching; however, angiographic assessment was unremarkable. Also, it is very unusual for any individual to twist, position, or stretch his or her own neck in an unusual posture, because such extreme neck movements would be limited by pain. It is hypothesized that in the absence of a potential structural finding that may have resulted in spinal contusion, there may be either a hyperextension or twisting injury causing a compressive myelopathy during self‐sustained stretching and anatomically abnormal maneuvers. This could be due to stenotic or mechanical cord compression only occurring in dynamic neck movements, which may not be evident on static imaging like MRI. The other possibility is that the radiological findings could be secondary to ischemic injury to the cord due to transient vascular flow abnormalities during extremes of neck movements. This could have been assessed by live fluoroscopic angiography with active movements of the neck; however, it would have put the patient at risk of a repeat insult.

Options for inpatient rehabilitation are fairly limited in Saudi Arabia, and early referral to comprehensive rehabilitation remains a considerable challenge.^9^ Functional recovery in patients with SCI may be affected due to delay in rehabilitation care.^10^ Our patient could have been referred to a comprehensive rehabilitation facility directly from the acute care, which may have prevented complications of SCI. A young patient, who was otherwise independent and active premorbid, remained dependent in activities of daily living after SCI and developed debilitating spasticity. In a survey on 151 patients with SCI, arm and hand function had the highest preference over other functional preferences among patients.^11^ Even in patients with paraplegia, the arm and hand function remains a priority over walking. This may be due to their reliance on upper limbs to compensate for their activity limitations including holding gait aids and propelling a mobility device. This case is reported not only on account of rarity, but also highlights the importance of comprehensive intensive integrated rehabilitation program to improve functional outcomes in similar patients. To our knowledge, this is the first case report of a SCIWORA due to active neck stretching in an adult.

## 4 CONCLUSION

This report raises concerns on the possibility of SCI due to abnormal neck movements, such as abrupt or sustained stretching of the neck. This remains relevant and important due to a common practice among people to stretch their necks due to fatigue or pain. This brings attention to importance of ergonomic history in patients with nontraumatic SCI and to inquire if they have been performing such maneuvers in their past. Similar to traumatic and nontraumatic SCI of other etiologies, comprehensive inpatient rehabilitation can improve functional outcomes in patients with SCIWORA due to active neck stretching; however, this conclusion cannot be generalized based on a single case report.

## CONFLICT OF INTEREST

The authors declared no conflicts of interest.

## AUTHOR CONTRIBUTIONS

SU: involved in conceptualization and design of study. YAA, SST: collected the data. SU, AZQ, SST: involved in drafting. SU, AZQ, YAA: revisited critically. SU, AZQ: approved the final version.

## References

[1] Yip PK , Malaspina A . Spinal cord trauma and the molecular point of no return. Mol Neurodegener. 2012;7:6. 22315999 10.1186/1750-1326-7-6 PMC3299607

[2] Robert AA , Zamzami MM . Traumatic spinal cord injury in Saudi Arabia: a review of the literature. The Pan Afr Med J. 2013;16:104. 24876893 10.11604/pamj.2013.16.104.2902 PMC4033590

[3] Khan AA , Mahmood S , Saif T , Gul A . Spinal cord injury without radiographic abnormality (SCIWORA) in adults: a report of two cases. J Pak Med Assoc. 2017;67(8):1275‐1277. 28839319

[4] Szwedowski D , Walecki J . Spinal cord injury without radiographic abnormality (SCIWORA) ‐ clinical and radiological aspects. Polish J Radiol. 2014;79:461‐464. 10.12659/PJR.890944 PMC4262055 25505497

[5] Cheong HS , Hong BY , Ko YA , Lim SH , Kim JS . Spinal cord injury incurred by neck massage. Ann Rehabil Med. 2012;36(5):708‐712. 23185737 10.5535/arm.2012.36.5.708 PMC3503948

[6] Seo JG , Kim JY , Choi HJ . Acute myelopathy due to ruptured HNP in cervical OPLL patient ‐ case report. J Korean Soc Spine Surg. 2006;13(4):323‐326.

[7] Hsieh JH , Wu CT , Lee ST . Cervical intradural disc herniation after spinal manipulation therapy in a patient with ossification of posterior longitudinal ligament: a case report and review of the literature. Spine (Phila Pa 1976). 2010;35(5):E149‐E151. 20190620 10.1097/BRS.0b013e3181bee8a7

[8] Abdel‐Mannan O , Mahmud I . A patient presenting with intact sensory modalities in acute spinal cord ischemia syndrome: a case report. J Med Case Rep. 2011;5:31. 21269425 10.1186/1752-1947-5-31 PMC3224536

[9] Mahmoud H , Qannam H , Zbogar D , Mortenson B . Spinal cord injury rehabilitation in Riyadh, Saudi Arabia: time to rehabilitation admission, length of stay and functional independence. Spinal Cord. 2017;55(5):509‐514. 28139661 10.1038/sc.2016.165 PMC5568447

[10] Adams MM , Hicks AL . Spasticity after spinal cord injury. Spinal Cord. 2005;43(10):577. 15838527 10.1038/sj.sc.3101757

[11] Lo C , Tran Y , Anderson K , Craig A , Middleton J . Functional priorities in persons with spinal cord injury: using discrete choice experiments to determine preferences. J Neurotrauma. 2016;33(21):1958‐1968. 27080545 10.1089/neu.2016.4423

